# Neuromagnetic speech discrimination responses are associated with reading-related skills in dyslexic and typical readers

**DOI:** 10.1016/j.heliyon.2020.e04619

**Published:** 2020-08-25

**Authors:** A. Thiede, L. Parkkonen, P. Virtala, M. Laasonen, J.P. Mäkelä, T. Kujala

**Affiliations:** a Cognitive Brain Research Unit, Department of Psychology and Logopedics, Faculty of Medicine, University of Helsinki, Finland; b Department of Neuroscience and Biomedical Engineering, School of Science, Aalto University, Finland; c Aalto Neuroimaging, Aalto University, Finland; d Department of Psychology and Logopedics, Faculty of Medicine, University of Helsinki, Finland; e Department of Phoniatrics, Helsinki University Hospital, Finland; f BioMag Laboratory, HUS Medical Imaging Center, Helsinki University Central Hospital, Finland

**Keywords:** Dyslexia, Magnetoencephalography (MEG), Speech processing, Mismatch field (MMF), Reading skills, Verbal working memory, Behavioral neuroscience, Cognitive neuroscience, Applied linguistics, Clinical psychology, Cognitive psychology

## Abstract

Poor neural speech discrimination has been connected to dyslexia, and may represent phonological processing deficits that are hypothesized to be the main cause for reading impairments. Thus far, neural speech discrimination impairments have rarely been investigated in adult dyslexics, and even less by examining sources of neuromagnetic responses. We compared neuromagnetic speech discrimination in dyslexic and typical readers with mismatch fields (MMF) and determined the associations between MMFs and reading-related skills. We expected weak and atypically lateralized MMFs in dyslexic readers, and positive associations between reading-related skills and MMF strength. MMFs were recorded to a repeating pseudoword /*ta-ta*/ with occasional changes in vowel identity, duration, or syllable frequency from 43 adults, 21 with confirmed dyslexia. Phonetic (vowel and duration) changes elicited left-lateralized MMFs in the auditory cortices. Contrary to our hypothesis, MMF source strengths or lateralization did not differ between groups. However, better verbal working memory was associated with stronger left-hemispheric MMFs to duration changes across groups, and better reading was associated with stronger right-hemispheric late MMFs across speech-sound changes in dyslexic readers. This suggests a link between neural speech processing and reading-related skills, in line with previous work. Furthermore, our findings suggest a right-hemispheric compensatory mechanism for language processing in dyslexia. The results obtained promote the use of MMFs in investigating reading-related brain processes.

## 1 Introduction

In developmental dyslexia, which is highly prevalent (up to 17%, Elliot and Grigorenko, 2014) and cumbersome for individuals in modern societies, reading-skill acquisition is compromised despite appropriate education and normal intelligence (Lyon et al., 2003). Dyslexia has been associated with significant difficulties in phonological processing (Laasonen et al., 2010; Ramus, 2001; Ramus et al., 2018), which may result from poor phonological representations or their accessibility (Ramus, 2001; Ramus et al., 2013). A range of other dysfunctions of cognition, such as deficits in working memory, especially verbal short-term memory, are associated with or potentially underlie reading deficits (Banai and Ahissar, 2004; Laasonen et al., 2009).

Dyslexia has been associated with several structural and functional brain abnormalities relevant for speech and language processing (Eckert et al., 2016; Giraud and Poeppel, 2012; Lehongre et al., 2011; Linkersdörfer et al., 2012; Richlan et al., 2013, 2011; 2009; Schulte-Körne et al., 2001). Acoustic–phonological processes pertinent for speech functions can be investigated with mismatch negativity (MMN) responses recorded by electroencephalography (EEG) or magnetoencephalography (MEG; Näätänen, 2001). MMN is an event-related component elicited by rare changes in a stream of repeating sounds (Näätänen et al., 1978), reflecting neural sound discrimination (Kujala and Näätänen, 2010). The primary MMN generators are located in bilateral temporal cortices (e.g., Alho, 1995). The left-hemisphere generator contributes more to speech processing than the right one, presumably reflecting native phonology (e.g., Näätänen et al., 1997; Shestakova et al., 2002; Shtyrov et al., 2000).

MMN is linked with language and reading skills, which makes it a promising neural marker for dyslexia. For example, larger MMN amplitudes to speech-sound changes have been associated with better phoneme processing skills in typically developing prereaders (Linnavalli et al., 2017) and better scores in pseudoword reading in children with auditory processing disorder (Sharma et al., 2006). This response can also predict future development, as shown by MMNs recorded to speech sounds in kindergarten (Maurer et al., 2009) and in infancy (Van Zuijen et al., 2013) that are associated with language and reading outcomes at school.

In line with this, MMNs to speech-sound and non-speech-sound changes were shown to be diminished and delayed in children and adults with dyslexia (for reviews, see Hämäläinen et al., 2013; Kujala and Näätänen, 2001; Schulte-Körne and Bruder, 2010), and even in infants and children having a familial risk of dyslexia (Benasich et al., 2006; Leppänen et al., 2002; Lovio et al., 2010; Schaadt et al., 2015; Schaadt and Männel, 2019; Schulte-Körne et al., 1998; Thiede et al., 2019; van Leeuwen et al., 2008; for a review, see Ozernov-Palchik and Gaab, 2016). Some studies, however, showed abnormally enhanced MMNs to sound changes in dyslexics (Corbera et al., 2006; Hämäläinen et al., 2008) and in others the MMNs were atypical in dyslexics only for certain stimulus types (Baldeweg et al., 1999; Meng et al., 2005; Schulte-Körne et al., 1998).

The language and speech processing deficits in reading impairments may also be reflected in atypical cerebral lateralization of these functions (Heim et al., 2004; Vandermosten et al., 2013; Zhao et al., 2016; however, see Wilson and Bishop, 2018). MMNs to tone or tone-pattern changes were found to be abnormally lateralized in dyslexia (Kujala et al., 2003; Kujala et al., 2000; Renvall and Hari, 2003; Sebastian and Yasin, 2008; see, however, Kujala et al., 2006; Schulte-Körne et al., 1999, 2001; Sharma et al., 2006). However, only few studies have investigated lateralization of speech-elicited MMNs in dyslexia or at-risk groups, with mixed results. For example, one study found left-lateralized MMN to phoneme changes in kindergarten to predict good reading skills and right-lateralized MMN poor reading skills at school (Maurer et al., 2003), whereas other studies reported no lateralization differences in the speech-elicited MMN between dyslexic and control groups (Schulte-Körne et al., 2001; Sebastian and Yasin, 2008).

Very few MMN studies on dyslexia have so far used spatially accurate methods to determine response strengths or lateralization. One such method is MEG which has better spatial resolution but the same excellent temporal resolution than EEG. Renvall and Hari (2003), utilizing MEG, reported weaker left-hemispheric mismatch fields (MMFs, used here, also called magnetic mismatch negativity, MMNm, the magnetic equivalent of MMN) to tone frequency changes in adult dyslexic than non-dyslexic readers. To our knowledge, the only previous study comparing MMFs in dyslexic and control children to speech-sound changes (/*ba*/ vs. /*da*/) failed to find group differences (Paul et al., 2006). The present MEG study addresses this apparent niche in dyslexia research. We also refined the spatial accuracy of MEG by applying individual head models from anatomical MRIs for MMF source localizations. To our knowledge, this is the first study investigating MMFs to phonetic changes in dyslexic adults and determining their association with neurocognitive language-related measures.

The present study aimed to investigate neural speech-sound discrimination in dyslexia with spatially accurate source estimates, and its association with reading-related skills. To this end, we recorded MMFs to several speech-sound changes (vowel, vowel duration, syllable frequency) in a phonotactically legal pseudoword and compared their source strengths and latencies between typical and dyslexic readers. Furthermore, we used an extensive neuropsychological test battery tapping reading and related skills (phonological processing, working memory) as well as intelligence quotient (IQ) of the participants. Since earlier studies have shown diminished and delayed MMN amplitudes in dyslexia (for reviews, see Hämäläinen et al., 2013; Kujala and Näätänen, 2001; Schulte-Körne and Bruder, 2010), our first hypothesis was that the dyslexic group exhibits diminished and/or delayed MMF source amplitudes. Secondly, based on previous MMN/MMF studies with tone stimuli in dyslexia (e.g., Kujala et al., 2003; Renvall and Hari, 2003), we hypothesized that the MMFs of the dyslexic group are less lateralized to the left hemisphere. As left-hemispheric MMN generators presumably reflect native phonology (e.g., Näätänen et al., 1997; Shestakova et al., 2002; Shtyrov et al., 2000), we hypothesized that they are also relevant for reading skills. Specifically, the third hypothesis was that better outcomes in all three reading-relevant skills correlate with stronger MMF source amplitudes in both groups, predominantly in the left hemisphere for phonetic changes, i.e., for vowel duration and vowel identity changes.

## 2 Material and methods

### 2.1 Participants

Forty-three healthy Finnish participants (21 dyslexics, 22 controls) aged 19–45 years without history of neurological diseases participated in the study. The inclusion criteria for the dyslexic group were a diagnostic dyslexia statement (from a psychologist, special education teacher or similar), or a history of reading difficulties in childhood (see Section 2.2) combined with below-norm performance in either speed or accuracy (below one standard deviation from age-matched standardized control data, see Laasonen et al., 2010) in two or more reading subtests (word list reading, pseudoword list reading, text reading, Nevala et al., 2006). Inclusion criteria for control participants were no report of dyslexia or co-occurring language disorders confirmed by within-norm performance in speed and accuracy in at least two reading subtests. General exclusion criteria were an individualized school curriculum (i.e., individualized education program due to special education needs) or attention deficit disorders (see Section 2.2), oral language development problems indicative of developmental language disorder, performance IQ below 80, and metal in the body. Participants gave their written informed consent, and all procedures employed conformed to the Declaration of Helsinki. The Coordinating Ethics Committee (Hospital District of Helsinki and Uusimaa) approved the study protocol. The study has been pre-registered in clinicaltrials.gov (ID NCT02622360).

### 2.2 Questionnaires and neuropsychological test battery

All participants filled questionnaires before brain imaging measurements including the Finnish versions of The Adult Reading History Questionnaire (ARHQ; Laasonen et al., 2014) and The Adult ADHD Self-Report Scale (ASRS; Kessler et al., 2005). Questionnaires were paired with clinical diagnostic interviews that enquired about past and current reading difficulties and dyslexia in relatives. The interviews also included questions assessing the exclusion criteria, such as those concerning broader cognitive deficits, oral language development problems and attention-related deficits. The neuropsychological tests were grouped into domains of technical reading, phonological processing, working memory, and intelligence. Technical reading (as opposed to reading comprehension in text reading that was used for dyslexia assessments, see Section 2.1) was assessed with word-list reading and pseudoword-list reading (speed and accuracy, Nevala et al., 2006). Phonological processing was assessed with a Nonword span test, in which participants repeated a lengthening sequence of nonwords (span length, Laasonen, 2002), the Pig Latin test, in which participants had to change the first syllables between two pseudowords (e.g., kouta – mesi rebuilds to meuta – kosi, accuracy, Nevala et al., 2006), and rapid alternate stimulus naming, in which changing stimuli (colours, number, letters) had to be named fast and accurately (RAS, speed in second trial, Wolf, 1986). Working memory was assessed with the Wechsler Memory Scale III (WMS-III, Wechsler, 2008) including visual working memory (subtest Visual Series) and verbal working memory (Number Series). Intelligence was assessed with the Wechsler Adult Intelligence Scale IV (Wechsler, 2005) including verbal IQ (Similarities and Vocabulary), performance IQ (Block Design and Matrix Reasoning), and full IQ (all four previous subtests). Composite scores were computed for phonological processing and technical reading by converting the raw scores of all subtests to *z*-scores and averaging them, and for working memory functions by following the procedure advised in WMS-III.

**Figure 1:**
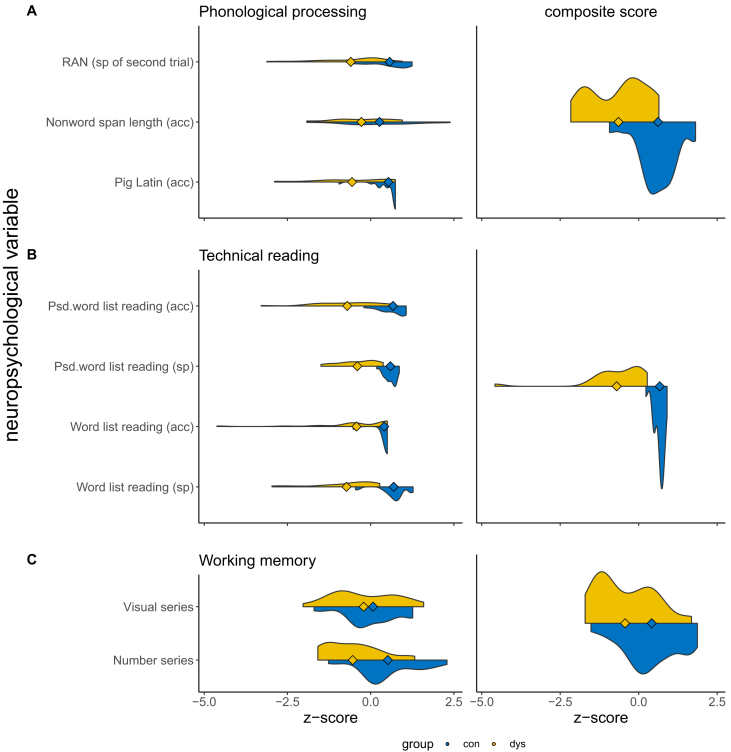
Neuropsychological profiles of the participants visualized by violin plots (blue – control group, yellow – dyslexic group). Means of group distributions are indicated by a diamond shape. Single variables are shown on the left and composite scores on the right panels. A) Phonological processing. B) Technical reading. C) Working memory. sp - speed, acc - accuracy, psd - pseudo.

The control group outperformed the dyslexic group in technical reading, phonological processing, working memory, as well as in verbal IQ, as expected (Table 1, Figure 1). However, the control group also had a higher performance IQ and higher education than the dyslexic group. As dyslexic readers are known to underperform in verbal, but not necessarily in performance IQ (Laasonen et al., 2009), the performance IQ was taken into account in the correlation analysis as a control variable, and analyses including group comparisons were repeated with groups matched for performance IQ (see 2.7).

**Table 1:** Demographic and neuropsychological characterization of the study groups.

Variable	CON (N = 22)	DYS (N = 21)	Comparison		
			test statistic	*p*	95% confidence interval
Demographic					
gender [f/m]	12/10	12/9	Χ^2^(1) = 0	1	
age [years]	29.9 (5.9)	31.0 (8.6)	*t*(35) = -0.48	.633	[-5.68 3.50]
education [years]	17.0 (2.5)	14.7 (2.5)	*t*(40) = 3.06	.004	[0.80 3.93]
music education^#^ [years]	0.25 (5.5)	0.0 (2.0)	W = 283.5	.152	

Neuropsychological					

full IQ	117.0 (7.0)	104.0 (9.4)	t(37) = 5.18	8.0∗10^−6^	[8.00 18.26]
verbal IQ^#^	115.0 (10.0)	100.0 (22.0)	W = 390.5	1.0∗10^−4^	
performance IQ	120.0 (10.0)	110.0 (12.3)	t(39) = 3.13	.003	[3.77 17.61]
phonological processing	0.49 (0.42)	-0.33 (0.60)	t(36) = 5.18	8.9∗10^−6^	[0.50 1.14]
technical reading^#^	0.61 (0.17)	-0.34 (0.82)	W = 461	3.8∗10^−12^	
working memory	24.3 (4.8)	19.8 (5.0)	t(41) = 3.01	.004	[1.49 7.53]

Notes. Mean and standard deviation (in brackets) for normally-distributed variables (Shapiro-Wilk test). Group comparison with independent-samples t-test. Median and interquartile range (in brackets) for non-normally-distributed variables, indicated by the hash sign^#^. Group comparison with Wilcoxon Rank Sum W-test. Test statistics, degrees of freedom (in brackets), significance levels *p*, and 95% confidence intervals are reported. CON – control group, DYS – dyslexic group.

### 2.3 Stimuli

The repetitive “standard” stimulus was a naturally recorded Finnish 300-ms-long pseudoword /*ta-ta*/ with the stress on the first syllable (Pakarinen et al., 2014; Thiede et al., 2019). Occasional “deviant” stimuli included a change in vowel duration (lengthening of second /*a*/ of the standard stimulus from 71 to 158 ms), vowel identity (adding /*o*/ to the second syllable from a natural recording of /*ta-to*/, pitch-controlled), or syllable frequency (shifting the f_0_ of the second syllable from 175 to 225 Hz) in the second syllable (edited with Adobe Audition CS6, 5.0, Build 708 and Praat 5.4.01). The duration of the vowel identity and syllable frequency deviants was identical to the duration of the standard stimulus. The intensity level of all deviants was root-mean-square normalized to match the average intensity level of the standard stimulus. The onset of change was at 180 ms from stimulus onset for the frequency and vowel deviants, and at 225 ms from stimulus onset for the duration deviant. Stimuli were presented pseudo-randomly (at least one standard always following a deviant) in two ≈12.6 min recording blocks, each containing 946 stimuli and starting with five standard stimuli. Standards were presented with a probability of approximately 75.3% and each deviant type with a probability of 8.3%. The stimulus-onset asynchrony (SOA) was 800 ± 50 ms (randomly alternating between 750, 760, 770, …, 840, 850 ms).

The stimuli were presented during MEG/EEG recordings with Presentation Software (Neurobehavioural Systems Ltd., Berkeley, CA, USA) binaurally via plastic tubes and silicon earphones at a comfortable level (≈70–80 dB SPL). During stimulation, participants were sitting, instructed to keep the head still and to attend to a self-selected, subtitled, and silenced movie projected (Panasonic PT-D7500E; Panasonic, Kadoma, Osaka, Japan) to a back-projection screen (MEGIN Oy, Helsinki, Finland) located 150 cm from the participant's head.

### 2.4 MEG/EEG and MRI procedure

MEG/EEG was recorded using a 306-channel Elekta Neuromag TRIUX (MEGIN Oy, Helsinki, Finland) whole-head MEG system (sampling rate 1 kHz and pass-band 0.03–330 Hz) in a magnetically shielded room (Euroshield/ETS Lindgren Oy, Eura, Finland) in BioMag Laboratory in Helsinki University Central Hospital (duration 2–3 h). Prior to the measurement, the positions of five head position indicator (HPI) coils and additional head surface points (EEG electrodes) were determined in relation to the nasion and both preauricular points with an Isotrak 3D-digitizer (Polhemus Inc., Colchester, USA). The head position with respect to the MEG sensor array was continuously monitored. Vertical and horizontal electro-oculograms (EOG) were recorded.

The anatomical T1-weighted images (MPRAGE) were acquired on a 3T MAGNETOM Skyra whole-body MRI scanner (Siemens Healthcare, Erlangen, Germany) with a 32-channel head coil at AMI centre of Aalto Neuroimaging (duration 30 min), Aalto University (176 slices, slice thickness 1 mm, voxel size 1 mm × 1 mm × 1 mm, field of view 256 mm × 256 mm). The images were checked for incidental findings by a physician.

### 2.5 Data preprocessing

The following processing steps were executed in MNE-Python software package v0.17.dev0 (Gramfort et al., 2014), unless indicated otherwise; the code is available at https://github.com/athiede13/neural_sources. Temporal signal space separation (tSSS; Taulu and Simola, 2006) with head movement compensation and interpolation of previously marked bad channels was performed with Maxfilter software (version 2.2.15; MEGIN Oy, Helsinki, Finland). Ocular and cardiac artifacts were removed by signal space projection (SSP; Tesche et al., 1995). The data were filtered to 0.5–30 Hz with a finite impulse response filter, and epochs were extracted 100 ms before and 840 ms after stimulus onset for all stimulus types for all channels and each participant and recording. Epochs with signal excursion exceeding 4 pT in magnetometers, 4 pT/cm in gradiometers and 250 μV in EOG channels were excluded from analysis. For the standard stimuli, on average 679 epochs (range 667–681) per participant were included in the analysis. For the frequency, duration, and vowel deviants, on average 144 (139–146), 145 (142–147), and 142 epochs (138–144), respectively, were included in the analysis per participant. Deviant-minus-standard subtraction curves (MMFs) were calculated for each deviant type with equal weights.

The anatomical MRI of each participant was preprocessed with Freesurfer software (http://surfer.nmr.mgh.harvard.edu/) version 5.3 and 6.0 following the standard procedure (Fischl, 2012). Manual editing of pial surface and white matter control points (64% and 18% of cases, respectively) ensured a correct segmentation of the cortex.

### 2.6 Source modeling

After MRI–MEG coregistration (*mne coreg*), the generators of individual MMFs were estimated in a cortically-constrained source space; the MEG forward solution was calculated for 4098 source points per hemisphere. The minimum-norm source estimate (MNE) was computed for the MMFs using depth-weighting (0.8), fixed-orientation constraint, and the 100-ms-pre-stimulus-baseline regularized noise covariance of pooled deviant and standard waveforms of each participant. The MNE source estimates were morphed to Freesurfer's average subject (*fsaverage*) cortical space and averaged for each group.

Auditory cortices (lateral sections of superior temporal gyrus and sulcus of the Destrieux Atlas, aparc.a2009s in Freesurfer, Destrieux et al., 2010) were *a priori* chosen as regions of interest (ROI) based on previous research (Alho, 1995; Renvall and Hari, 2003). As this region is considerably larger than the presumed region generating MMF, the average of the dipole moments within that ROI is not representative of the activity of interest. Therefore, data-driven functional ROIs were created based on the MNE source estimate of each participant for each MMF at the individual peak time within the MMF time window (see below). Specifically, the functional ROIs were created by taking the top 60% of individual peak source estimates within the anatomical *a priori* -defined ROI and then combined into one for the most consistent individual source points (in more than 45% of all functional ROIs) to represent the area with the most consistent activity across participants. The source time course at the functional ROI were extracted with source signs flipped depending on the source orientation (*pca_flip*) to avoid cancellation.

Time windows for determining the maximum MMF source amplitudes and latencies were selected around the visually inspected peaks of the averages across all participants and deviant types, groups combined, resulting in a 100-ms wide window for the first peak (120–220 ms after change onset for frequency and vowel deviant, 75–175 ms after change onset for duration deviant) and a 200-ms wide window for the second peak (270–470 ms after change onset for frequency and vowel deviant, 225–425 ms after change onset for duration deviant). Individual maximum amplitudes and the corresponding latencies were extracted within the determined time windows and ROI for statistical analysis in both hemispheres.

### 2.7 Statistical analysis

SPSS version 25.0.0.1 (IBM, Armonk, New York, USA), R (R Core Team, 2018) and RStudio version 1.1.453 (RStudio Team, 2016) were used for statistical analyses. To validate that MMFs were elicited in the selected time windows in both groups, MMF source amplitudes (difference waveforms) were tested for statistical significance with one-sample *t*-tests against zero separately in each group. Group and laterality effects and their interactions on MMF source amplitudes (difference waveforms) were analyzed with three-way repeated-measures analysis of variance (ANOVA) with group (control, dyslexic) as between-subjects factor and laterality (left, right), deviant (frequency, duration, vowel), and time (MMF, late MMF) as within-subjects factors. Group and laterality effects and their interactions on MMF source latencies were analyzed with two-way repeated-measures ANOVAs with group as between-subjects factor and laterality and deviant as within-subjects factors. In the ANOVAs, main effects of deviant or time were not investigated. Significant three-way interactions were further investigated with separate follow-up ANOVAs. Greenhouse-Geisser correction was applied when the sphericity assumption was violated. Bonferroni correction was used to account for multiple comparisons in all *post-hoc* tests and only corrected *p*-values are reported. Although performance IQ differed between the groups, an analysis of covariance with performance IQ as a covariate was not performed, following the recommendations of Dennis et al. (2009). Instead, to ensure that the IQ difference would not explain the results, statistical analyses comparing groups were repeated for a sample in which the groups were matched for performance IQ (*N* = 37, 19 in control group, 18 in dyslexic group). The profile of the matched groups did not otherwise differ from the original sample.

Partial Pearson correlations were computed between MMF source amplitudes and neuropsychological test scores, controlling for the effect of performance IQ. In order to reduce the amount of tests, correlations were analyzed in three steps both separately for the two groups and across groups; (1) for MMF source amplitudes at two hemispheres averaged across all deviants with the three neuropsychological composite scores, if significant, then (2) separately for MMFs to each deviant, if significant, then (3) separately for each subcomponent of the neuropsychological composite scores. Despite a potential risk of circular inference (Kriegeskorte et al., 2009), this stepwise procedure was chosen to output the most meaningful associations from a neuroscientific and neuropsychological perspective. Bonferroni correction for multiple comparisons was employed at each step, as recommended by Rousselet and Pernet (2012).

## 3 Results

**Figure 2:**
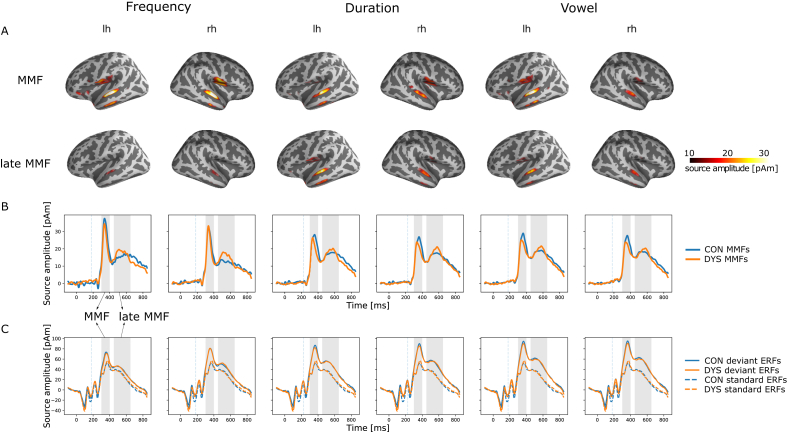
Neural sources of MMF and late MMF to frequency, duration, and vowel changes. A) Source locations as MNE's on lateral brain surfaces. B) Extracted ROI time courses (subtraction curves, i.e. MMFs and late MMFs). C) Event-related fields (ERFs) to standard and deviant (frequency, duration, and vowel deviants) stimuli extracted from the same ROI. Grey shaded areas in ROI time courses (B and C) depict the time windows of interest (first – MMF, second – late MMF) and dotted vertical lines represent the change onset. lh – left hemisphere, rh – right hemisphere, CON – control group, DYS – dyslexic group.

The MMF source waveform (difference waveform) indicated two responses (Figure 2), referred to as MMF and late MMF (peaking at 125–170 ms and 325–370 ms from change onset, respectively) that were both significantly larger than zero in both groups and for all deviants (amplitude range 15–44 pAm; Table 2). Their peak activations were located in the left middle temporal cortex (BA21 and BA22) and in the right superior temporal cortex (BA41 and BA22; Figure 2).

**Table 2:** Descriptive statistics (mean M, standard deviation SD) of maximal source amplitudes and one-sample t-test results for significance testing against zero (with confidence interval CI).

Deviant	Group	Hemi	Descriptives		One-sample *t*-test			
			*M* [pAm]	*SD* [pAm]	*t*	*df*	*p*	95% *CI*
MMF								
fre	con	lh	44	20	10.04	21	<.001	[35 53]
		rh	33	17	9.21	21	<.001	[26 41]
	dys	lh	39	14	12.92	20	<.001	[32 45]
		rh	36	20	8.30	20	<.001	[27 45]
dur	con	lh	38	22	8.29	21	<.001	[29 48]
		rh	31	11	13.90	21	<.001	[26 36]
	dys	lh	26	11	10.58	20	<.001	[21 32]
		rh	30	18	7.76	20	<.001	[22 38]
vow	con	lh	42	21	9.15	21	<.001	[32 51]
		rh	26	14	9.18	21	<.001	[20 32]
	dys	lh	37	14	11.87	20	<.001	[31 44]
		rh	25	15	7.98	20	<.001	[19 32]

late MMF								
fre	con	lh	23	17	6.59	21	<.001	[16 31]
		rh	15	20	3.64	21	.002	[7 24]
	dys	lh	24	12	9.41	20	<.001	[19 30]
		rh	20	20	4.50	20	<.001	[11 29]
dur	con	lh	37	10	17.81	21	<.001	[33 41]
		rh	25	14	8.54	21	<.001	[19 31]
	dys	lh	32	14	10.25	20	<.001	[26 39]
		rh	26	15	8.06	20	<.001	[20 33]
vow	con	lh	33	14	11.23	21	<.001	[27 39]
		rh	22	18	5.70	21	<.001	[14 30]
	dys	lh	32	10	14.46	20	<.001	[27 36]
		rh	20	18	4.95	20	<.001	[11 28]

Full statistics of significant effects in all ANOVAs and *post-hoc* analyses are reported in Table 3. A significant main effect was found for laterality, indicating larger left- than right-hemispheric responses. A significant two-way interaction effect was found between laterality and deviant, as well as time and deviant. A significant three-way interaction effect between laterality, deviant, and time was also found for the MMF source amplitudes (difference waveforms). A follow-up ANOVA for the MMF time windows revealed a significant laterality main effect, indicating larger left- than right-hemispheric responses. A significant interaction effect was found between laterality and deviant for the MMF time window. *Post-hoc* analysis of that interaction indicated larger left- than right-hemispheric MMFs to the vowel deviant. A follow-up ANOVA for the late MMF time window revealed a significant main effect of laterality, indicating larger left- than right-hemispheric responses.

**Table 3:** Significant ANOVA effects (N = 43).

		Model	ANOVA						*post-hoc*						
			effect	*F*	*df1*	*df2*	*p*	$\eta_{p}^{2}$	effect	*EMM1*	*(SEM1)*		*EMM2*	*(SEM1)*	*p*
AMPLITUDES		laterality ∗ deviant ∗ time ∗ group	laterality	9.48	1.00	41	.004	.19	left > right	34	(2)	>	26	(2)	
			time	30.49	1.00	41	<.001	.43	MMF > late MMF	34	(2)	>	26	(1)	
			laterality ∗ deviant	5.44	2.00	82	.006	.12	not tested, due to three-way interaction effect						
			deviant ∗ time	39.52	1.65	82	<.001	.49	not tested, due to three-way interaction effect						
			laterality ∗ deviant ∗ time	4.75	2.00	82	.011	.10	separate ANOVAs for the two time windows (below), due to several significant pairwise comparisons						
	MMF	laterality ∗ deviant ∗ group	laterality	6.89	1.00	41	.024	.14	left > right	38	(2)	>	30	(2)	
			deviant	9.03	2.00	82	.001	.18	not tested, due to interaction effect						
			laterality ∗ deviant	7.91	2.00	82	.001	.16	vow: left > right	40	(3)	>	26	(2)	<.001
									left: fre > dur	41	(3)	>	32	(3)	<.001
									left: vow > dur	40	(3)	>	32	(3)	.003
									right: fre > vow	35	(3)	>	26	(2)	.003
	late MMF	laterality ∗ deviant ∗ group	laterality	9.41	1.00	41	.008	.19	left > right	30	(2)	>	21	(2)	.004
			deviant	14.98	2.00	82	<.001	.27	fre < dur	21	(2)	<	30	(2)	<.001
									fre < vow	21	(2)	<	27	(2)	.005
LATENCIES	MMF	laterality ∗ deviant ∗ group	deviant	34.65	2.00	82	<.001	.46	fre < dur	340	(2)	<	368	(3)	<.001
									fre < vow	340	(2)	<	360	(3)	<.001
	late MMF	laterality ∗ deviant ∗ group	deviant	7.59	2.00	82	.001	.16	fre < dur	534	(6)	<	561	(7)	.007
									vow < dur	534	(6)	<	561	(7)	.011
			laterality ∗ group	4.46	1.00	41	.041	.10	con: left > right	555	(10)	>	526	(8)	.010

Notes: *p*-values are Bonferroni-corrected for all *post-hoc* analyses, i.e., for separate ANOVAs for the two time windows (two latter ANOVAs for amplitudes) and all *p*-values on the right-most column. EMM and SEM for analyses referring to amplitudes are in pAm and for analyses referring to latencies in ms. fre – frequency, dur – duration, vow – vowel, con – control group, $\eta_{p}^{2}$*–* effect size (partial eta squared), EMM – estimated marginal means, SEM – standard error of means.

For the late MMF source latencies, a significant interaction effect between laterality and group was found, indicating slower late MMFs in the left than right hemisphere in the control group only. The reported ANOVA results remained similar when repeating the analyses for performance-IQ-matched subsamples of control and dyslexic groups; only the latter effect on late MMF latencies disappeared (Table 4).

**Table 4:** Significant ANOVA effects (N = 37).

		Model	ANOVA						Post-hoc						
			effect	*F*	*df1*	*df2*	*p*	$\eta_{p}^{2}$	effect	*EMM1*	*(SEM1)*		*EMM2*	*(SEM1)*	*p*
AMPLITUDES		laterality ∗ deviant ∗ time ∗ group	laterality	6.90	1.00	35	.013	.16	left > right	34	(2)	>	26	(2)	
			time	36.42	1.00	35	<.001	.51	MMF > late MMF	34	(2)	>	25	(2)	
			laterality ∗ deviant	4.56	2.00	70	.014	.12	not tested, due to three-way interaction effect						
			deviant ∗ time	34.69	1.66	70	<.001	.50	not tested, due to three-way interaction effect						
			laterality ∗ deviant ∗ time	4.97	2.00	70	.010	.12	separate ANOVAs for the two time windows (below), due to several significant pairwise comparisons						
	MMF	laterality ∗ deviant ∗ group	deviant	7.51	2.00	70	.002	.18	not tested, due to interaction effect						
			laterality ∗ deviant	7.49	2.00	70	.002	.18	vow: left > right	40	(3)	>	26	(2)	<.001
									left: fre > dur	42	(3)	>	32	(3)	<.001
									left: vow > dur	40	(3)	>	32	(3)	.004
									right: fre > vow	35	(3)	>	26	(2)	.008
	late MMF	laterality ∗ deviant ∗ group	laterality	7.09	1.00	35	.023	.17	left > right	30	(2)	>	21	(3)	.012
			deviant	13.65	2.00	70	<.001	.28	fre < dur	20	(2)	<	29	(2)	<.001
									fre < vow	20	(2)	<	26	(2)	.002
LATENCIES	MMF	laterality ∗ deviant ∗ group	deviant	2.00	70.00	30	<.001	.46	fre < dur	340	(2)	<	368	(3)	<.001
									fre < vow	340	(2)	<	360	(3)	<.001
	late MMF	laterality ∗ deviant ∗ group	deviant	2.00	70.00	7	.001	.17	fre < dur	533	(7)	<	562	(8)	.009
									vow < dur	534	(7)	<	562	(8)	.017

Notes: *p*-values are Bonferroni-corrected for all *post-hoc* analyses, i.e., for separate ANOVAs for the two time windows (two latter ANOVAs for amplitudes) and all *p*-values on the right-most column. EMM and SEM for analyses referring to amplitudes are in pAm and for analyses referring to latencies in ms. fre – frequency, dur – duration, vow – vowel, con – control group, $\eta_{p}^{2}$*–* effect size (partial eta squared), EMM – estimated marginal means, SEM – standard error of means.

**Figure 3:**
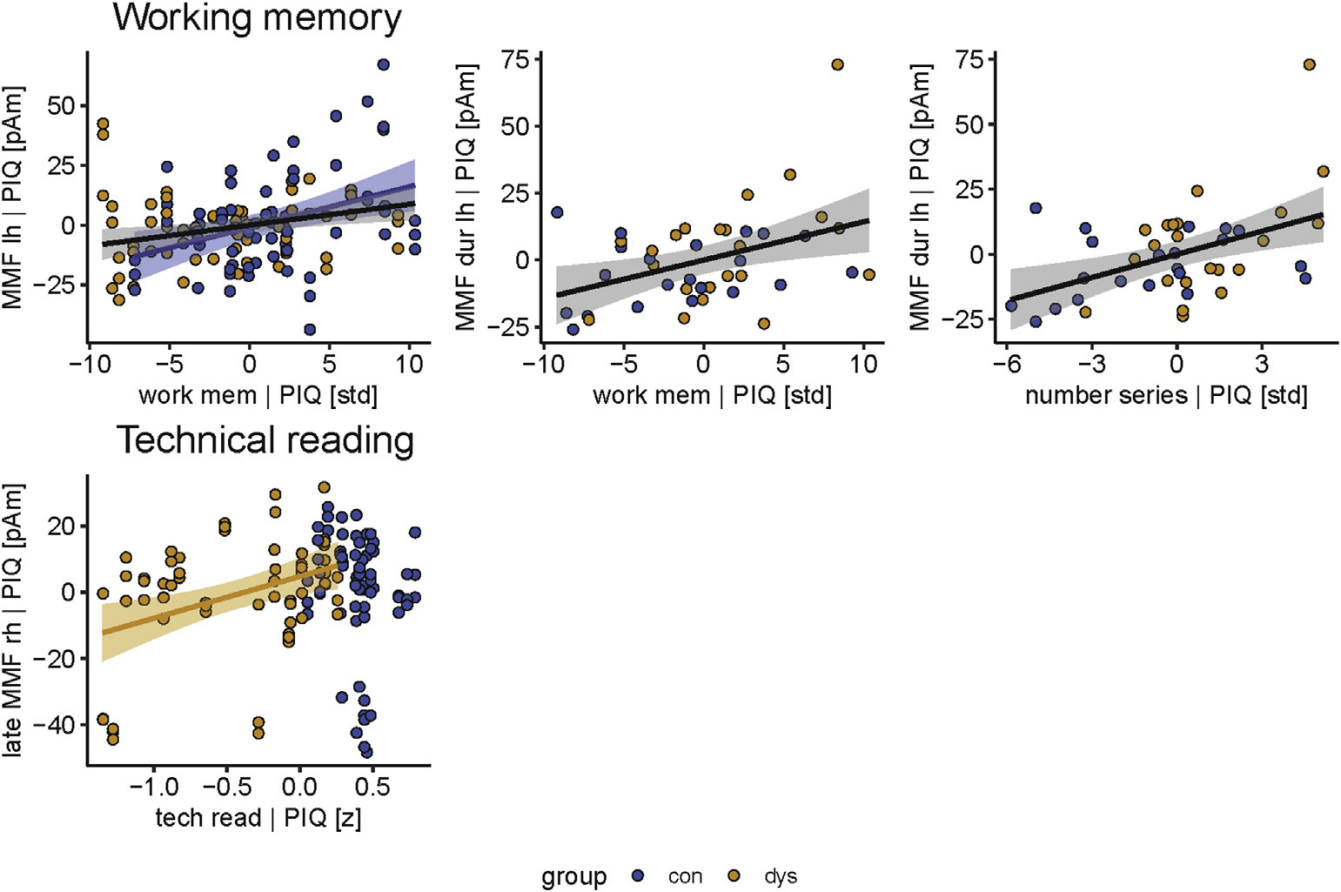
Significant partial Pearson correlations after Bonferroni correction of MMF and late MMF amplitudes with reading-related skills controlled for performance IQ. Scatter plots with linear regression lines (black – across both groups, blue – control group, yellow – dyslexic group) are shown for both all deviants pooled together (upper left and bottom panel) and separately for one deviant (upper middle and right panels). Number series is one subcomponent of the working memory composite score (upper right panel). One outlier (dyslexic) was removed, because of the technical reading score being below three interquartile ranges. work mem – working memory, tech read – technical reading, lh – left hemisphere amplitude, rh – right hemisphere amplitude, PIQ – performance IQ.

Source amplitudes of MMF and late MMF correlated with neuropsychological test scores (Figure 3, Table 5). Larger MMF source amplitudes in the left hemisphere were weakly correlated (r = .25; p = .03) with better working memory skills across all deviants and across both groups. Separate correlation tests for the deviants revealed a moderate correlation across groups for the left duration MMF with working memory skills (r = .40; p = .024). Separate working memory subtest analysis (visual and verbal) only showed a significant moderate correlation between the left duration MMF and the verbal working memory component (r = .46; p = .006). Within the control group, left MMFs across all deviants correlated moderately (r = .37; p = .015) with working memory skills. In separate tests for the deviants, the left MMF for the duration deviant was significant when uncorrected, but did not remain significant after Bonferroni correction. Within the dyslexic group, larger right MMFs (only uncorrected) and right late MMFs (also significant after corrections) across all deviants correlated moderately strongly (r = .36; p = .028) with better technical reading skills. In separate tests for the deviants, none remained significant.

**Table 5:** Partial Pearson correlations of MMF source amplitudes with neuropsychological tests controlled for performance IQ.

	comp.	deviant	correlation	*p*_*uncorr*_	*p*_*corr*_	*signif.*	*r*
both groups pooled (N = 43)	**1a) correlations of lh & rh amplitudes for all deviants pooled with three composite scores (*df* = 126)**						
	MMF	all	lh ~ phon	0.058	0.350		0.17
			rh ~ phon	0.441	2.648		-0.07
			lh ~ read	0.110	0.657		0.14
			rh ~ read	0.327	1.962		0.09
			**lh ~ work mem**	**0.005**	**0.030**	**∗**	**0.25**
			rh ~ work mem	0.832	4.995		-0.02
	late MMF	all	lh ~ phon	0.429	2.573		0.07
			rh ~ phon	0.782	4.692		0.02
			lh ~ read	0.311	1.864		0.09
			rh ~ read	0.057	0.341		0.17
			lh ~ work mem	0.111	0.665		0.14
			rh ~ work mem	0.223	1.336		0.11
	**1b) correlations of lh amplitudes for deviants separately with working memory (*df* = 40)**						
	MMF	frequency	lh ~ work mem	0.429	1.287		0.13
		**duration**	**lh ~ work mem**	**0.008**	**0.024**	**∗**	**0.40**
		vowel	lh ~ work mem	0.156	0.469		0.22
	**1c) correlations of lh amplitudes of duration MMF with subcomponents of working memory (*df* = 40)**						
	**MMF**	**duration**	**lh ~ number series**	**0.002**	**0.006**	**∗∗**	**0.46**
			lh ~ visual series	0.117	0.352		0.25
control group (N = 22)	**2a) correlations of lh & rh amplitudes for all deviants pooled with three composite scores (*df* = 63)**						
	MMF	all	lh ~ phon	0.011	0.069		0.31
			rh ~ phon	0.407	2.439		0.10
			lh ~ read	0.051	0.307		-0.24
			rh ~ read	0.209	1.256		-0.16
			**lh ~ work mem**	**0.003**	**0.015**	**∗**	**0.37**
			rh ~ work mem	0.327	1.963		0.12
	late MMF	all	lh ~ phon	0.860	5.159		0.02
			rh ~ phon	0.822	4.931		0.03
			lh ~ read	0.124	0.745		-0.19
			rh ~ read	0.491	2.947		-0.09
			lh ~ work mem	0.085	0.508		0.22
			rh ~ work mem	0.247	1.480		0.15
	**2b) correlations of lh amplitudes for deviants separately with working memory (*df* = 19)**						
	MMF	frequency	lh ~ work mem	0.122	0.367		0.35
		duration	lh ~ work mem	0.037	0.111		0.46
		vowel	lh ~ work mem	0.169	0.507		0.31
dyslexic group (N = 21)	**3a) correlations of lh & rh amplitudes for all deviants pooled with three composite scores (*df* = 60)**						
	MMF	all	lh ~ phon	0.843	5.058		-0.03
			rh ~ phon	0.164	0.986		-0.18
			lh ~ read	0.155	0.928		0.19
			rh ~ read	0.042	0.253		0.27
			lh ~ work mem	0.770	4.622		0.04
			rh ~ work mem	0.440	2.642		-0.10
	late MMF	all	lh ~ phon	0.455	2.728		0.10
			rh ~ phon	0.909	5.452		-0.01
			lh ~ read	0.436	2.615		0.10
			**rh ~ read**	**0.005**	**0.028**	**∗**	**0.36**
			lh ~ work mem	0.969	5.813		0.01
			rh ~ work mem	0.894	5.364		0.02
	**3b) correlations of rh amplitudes for deviants separately with technical reading (*df* = 17)**						
	late MMF	frequency	rh ~ read	0.059	0.178		0.44
		duration	rh ~ read	0.251	0.752		0.28
		vowel	rh ~ read	0.110	0.329		0.38

Notes. Reported are partial Pearson correlations. Row is **bolded,** when Bonferroni-corrected significance levels are at *p*_*corr*_ < .05. One outlier (dyslexic) was removed, because of the technical reading score being below three interquartile ranges. lh – left hemisphere amplitude, rh – right hemisphere amplitude, phon – phonological processing skills, read – technical reading score, work mem – working memory score, acc – accuracy.

## 4 Discussion

Our goals were to determine whether neural speech-sound discrimination is deficient or abnormally lateralized in adult dyslexic readers, and whether the speech-elicited neural responses correlate with reading-related skills. To improve spatial accuracy from previous EEG studies we recorded MMFs with MEG, and utilized individual MRIs for source localization. We found MMFs and late MMFs from bilateral auditory cortices to all three speech-sound changes. Furthermore, the MMFs and late MMFs were left-lateralized to all three deviants. For the MMFs, the effect was driven by the vowel deviant. Contrary to our expectations, MMFs and late MMFs did not differ in source amplitudes, latencies, or lateralization between dyslexic and typical readers. However, the MMFs were associated with skills pertinent for reading that are known to be affected by dyslexia (D'Mello and Gabrieli, 2018). Correlations were found between stronger left-hemispheric MMFs to the duration deviant and better verbal working memory skills in both groups pooled, and between stronger right-hemispheric late MMFs across deviants and more accurate and faster reading in the dyslexic group. This highlights the functional role of speech-related brain activity in reading and its impairments and promotes the utilization of the auditory MMF as a potential neural marker of abnormal reading.

### 4.1 MMF, late MMF and speech-sound discrimination

Two response components were found: The MMF peaked at around 125–170 ms after change onset with a clear and narrow peak. This latency is well within the established time range of the MMN/MMF (Kujala et al., 2007). An additional broader, smaller response (late MMF) peaked at around 325–370 ms. A similar response, the late MMN, has been reported in children (Cheour et al., 2001; Volkmer and Schulte-Körne, 2018), but rarely in adults (around 340–600 ms; Hill et al., 2004; Hommet et al., 2009; Korpilahti et al., 1995; Schulte-Körne et al., 2001; Zachau et al., 2005). Its functional role is still poorly understood. It was proposed to reflect linguistic processes as it was elicited by vowel but not by tone changes (Hill et al., 2004; Korpilahti et al., 1995). Late MMNs can, however, be elicited by simple and complex tone changes as well (Schulte-Körne et al., 2001; Zachau et al., 2005). The associations obtained between stronger late MMF in the right hemisphere and more accurate and faster reading-related skills in the dyslexic group support its relevance for linguistic processes in dyslexia. However, the nature of this response remains to be investigated in more detail by future studies.

The MMFs and late MMFs in both groups originated from primary and secondary auditory cortices (peak MNI coordinates corresponded to BAs 41, 21, 22), confirming the findings of previous MMN localization studies applying other types of source modeling (Alho, 1995; Escera et al., 2000). Furthermore, the obtained left-hemispheric lateralization of MMFs to vowel deviants is in line with previous MMF studies on speech processing (e.g., Näätänen et al., 1997; Shtyrov et al., 2000). Late MMFs in the current study were left-lateralized to all three deviants. To our knowledge there are no source-level studies on the late MMF to speech sounds. Therefore, a more accurate role of this response in speech or generally in sound processing remains to be determined with future studies employing speech and non-speech stimuli.

### 4.2 Group comparisons of MMF source strengths and latencies

We expected to find diminished MMF source amplitudes, less prominent lateralization to the left hemisphere, and delayed latencies in dyslexic than typical readers, but no significant group differences emerged. These results contradict with many previous studies, which have shown diminished MMNs to speech-sound changes in dyslexia or dyslexia risk (Schulte-Körne and Bruder, 2010, for a review). The only one previous study comparing MMFs in dyslexic and control participants to speech-sound changes (/*ba*/ vs. /*da*/) also failed to find group differences (Paul et al., 2006). The authors suggested that this could have resulted from a too large stimulus difference that was too easy to discriminate for their dyslexic children. This is consistent with previous observations showing diminished MMNs in dyslexia for small but not for large stimulus differences in tone frequency (Baldeweg et al., 1999). The same could be one reason for insignificant MMF source strength differences between the groups in our study.

Alternatively or additionally, the dyslexic subsamples of the different studies might differ in terms of their phonological deficits. An elaboration of the phonological deficit theory (Bradley and Bryant, 1983; Ramus, 2001; Ramus et al., 2013; Snowling, 2000) suggests that rather than the phonological representations per se, access to them may be impaired in dyslexia (Boets et al., 2013; Ramus and Szenkovits, 2008). The access of phonological representations is required for the Pig Latin and rapid naming subtests of the phonological processing composite, in which dyslexics of this study underperformed. This result combined with the present normal-like MMFs to speech-sound changes in our dyslexic group suggests that they had normal-like, but poorly accessible phonological representations. Future studies should determine the prevalence of core phonological deficits vs. dysfunctions in accessing or associating phonemes during reading in dyslexia in large participant samples. For instance, it was shown that dyslexics who displayed normal-like MMNs to phoneme changes presented with meaningless visual stimuli had diminished responses to the same changes when they were accompanied with written input (Mittag et al., 2013). Possibly, a larger proportion of dyslexics suffer from impairments in integrating and accessing of phonological information than merely from their poor representations.

In our previous study (Thiede et al., 2019) utilizing identical stimuli and paradigm as the current one, we found absent and atypical MMNs in infants at risk of dyslexia. This is quite a robust finding, since only ≈40–70% of children at risk of dyslexia become reading impaired (DeFries and Alarcón, 1996). The absence of such deficits in adult dyslexics suggests that neurobiological abnormalities in dyslexia might be more disruptive in infancy/childhood than in adulthood (see also, e.g., Lovio et al., 2010, reporting diminished MMNs to a range of speech stimuli in at-risk children). Possibly, speech development is originally delayed in dyslexia but speech processes become more normal by adulthood (Galaburda et al., 2006).

### 4.3 Correlation of MMF source strengths with reading-related skills

We also determined whether reading-related measures are associated with MMF source strengths. We found that larger MMF source amplitudes in the left hemisphere were associated with better working memory skills across both groups. *Post-hoc* analyses showed that the association was mainly driven by the MMF to the duration deviant and the verbal component of working memory. The result is in line with our hypothesis on positive correlations between MMF strengths and reading-related skills, and is consistent with previous studies that have shown associations between verbal working memory and MMN (Čeponienė et al., 1999; Watson et al., 2007) and late discriminative negativity (Hämäläinen et al., 2015), the equivalent of late MMN in children (LDN, Cheour et al., 2001). For example, children with increased verbal working memory performance had a larger MMN to consonant changes in speech sounds and tone frequency changes (Čeponienė et al., 1999; Watson et al., 2007). Yet, another study found no connection between the MMN and working memory in adults (Light et al., 2007). Compared to previous studies, our sample sizes are larger and we applied strict corrections for type I errors, which makes the findings more robust. The links others and we found between verbal working memory and neural speech discrimination suggest that accurate and efficient early stages of neural speech discrimination are paralleled by better verbal working memory performance. Working memory impairments especially in the phonological domain can delay or hamper language development in children (Adams and Gathercole, 2000). The phonological component of working memory has been linked with speech perception in noise. Working memory could solve mismatches, when noisy speech input and existing phonological representations are compared during speech processing (Millman and Mattys, 2017). This could also be a relevant mechanism during speech processing in everyday noisy conditions.

Even though this relationship between working memory and MMF source amplitudes was found across groups and in the control group, a separate analysis for the dyslexic group yielded no significant effects. Possibly, the proposed connection between automatic speech processing and working memory could be disrupted in dyslexia, in which particularly verbal working memory problems are relatively common (Banai and Ahissar, 2004; Laasonen et al., 2009). Further investigations on the connections between working memory, reading, and speech processing are needed in order to better understand their role and interplay also in dyslexia.

An association was also found between the MMF strength and technical reading in the dyslexic group: consistent with our hypothesis, larger late MMFs were correlated with better reading skills. It is notable that the lack of this association in the control group could result from the lack of variation in the technical reading scores due to a ceiling effect (see Figure 1). The association of the late MMF to reading skills was similarly shown in children, i.e., increased left-hemispheric late MMN/LDN was associated with better word-reading skills (Maurer et al., 2009). The LDN has also shown associations to verbal working memory in children (Hämäläinen et al., 2015). The authors suggested that the LDN may reflect further processing of the speech-sound changes and/or attention-related processes relevant for reading. This could be the case also for the late MMF in the present study: it may reflect more complex neural processes than the MMF that may be more relevant for language and reading which has also been proposed in earlier studies (Hill et al., 2004; Korpilahti et al., 1995). The finding of correlations between late MMF and reading in our dyslexic group only emerging in the right hemisphere is novel. Other neurophysiological responses in the right hemisphere, such as enhanced ERPs to pseudowords and higher EEG correlation indices, have previously been associated with reading skills in dyslexic or reading impaired children (Byring et al., 2004; Lohvansuu et al., 2014). Our results might, therefore, suggest that some dyslexics have developed a right-hemispheric compensatory mechanism for speech processing that is also beneficial for their reading skills (Eden et al., 2004; Sebastian and Yasin, 2008).

### 4.4 Limitations

The following limitations of the present study should be considered. The first one relates to the selection of the study sample. Despite attempts to find matching groups of dyslexics and typical readers, typical readers still were more educated and had higher PIQ in our sample compared to the dyslexics. We addressed this by repeating our analysis with a performance-IQ -matched subsample and obtained similar results. Group sizes in this study were higher than in most previous studies, but still moderate consisting of 20+ participants in each group. Larger-scale studies should be carried out, as the neuroimaging field suffers from replication failures of previous results obtained with small sample sizes (Kellmeyer, 2017). As the expected effect sizes are generally small, many of these studies might be underpowered. Second, the stimuli chosen for the current study might not be sufficiently sensitive to reveal phonological deficits in adult dyslexics, since diminished MMNs have mostly been reported for consonant changes (Noordenbos et al., 2013; Schulte-Körne et al., 2001; Tuomainen, 2015).

The present study was not designed to compare speech- vs. non-speech processing and the influence of dyslexia on this processing, which has been a long-debated issue in the literature (see, e.g., Schulte-Körne and Bruder, 2010). Therefore, non-speech stimuli were not included in our experimental paradigm, and we cannot exclude the possibility that our findings also reflect basic auditory processes instead of, or in addition to, speech-specific processes. However, the left-hemispheric lateralization of MMFs in the present study is compatible with previous studies on speech processing, as pointed out earlier (Section 4.1).

### 4.5 Conclusions

To summarize, our results, advanced with source-localization constraints from individual anatomical brain images, support the suggestion of bilateral sources of the MMF to speech-sound changes in auditory cortices, as well as left-hemispheric lateralization of the MMF to vowel changes and well as late MMF to frequency, vowel, and vowel duration changes. We found comparable MMF strengths, latencies, and lateralization in typical and dyslexic readers, not supporting the proposed abnormalities in neural speech-sound discrimination in dyslexia. Possibly our stimuli were not sensitive enough to probe these deficiencies, or our participant subsample did not predominantly have phonological representation problems. However, we found correlations between the MMFs to speech-sound changes and reading-related skills, highlighting the connection of neural low-level speech processing and reading in adults, and promoting the use of MMFs in investigating reading-related brain processes.

## Declarations

### Author Contribution

A. Thiede: Conceived and designed the experiments; Performed the experiments; Analyzed and interpreted the data; Contributed reagents, materials, analysis tools or data; Wrote the paper.

L. Parkkonen: Analyzed and interpreted the data; Contributed reagents, materials, analysis tools or data; Wrote the paper.

P. Virtala, T. Kujala: Conceived and designed the experiments; Analyzed and interpreted the data; Wrote the paper.

M. Laasonen: Conceived and designed the experiments; Contributed reagents, materials, analysis tools or data; Wrote the paper.

J.P. Mäkelä: Contributed reagents, materials, analysis tools or data; Wrote the paper.

### Funding statement

This work was supported by 10.13039/501100004012Jane and Aatos Erkko Foundation, Academy of Finland [project numbers 2764141 and 316970], KELA (The Finnish Social Insurance Institution), and University of Helsinki MRI measurement support. Anja Thiede was supported by the University of Helsinki Research Foundation and Arvo and Lea Ylppö foundation.

### Competing interest statement

The authors declare no conflict of interest.

### Additional information

The clinical trial described in this paper was registered at ClinicalTrials.gov under the registration number NCT02622360.
